# Age Effect on the Bidirectional Relations Between Alcohol Consumption and Pain

**DOI:** 10.1093/geroni/igab046.1115

**Published:** 2021-12-17

**Authors:** Ellen Yeung, Matthew Lee, Kenneth Sher

**Affiliations:** 1 George Washington University, Washington, District of Columbia, United States; 2 Rutgers University, Piscataway, New Jersey, United States; 3 University of Missouri, Columbia, Columbia, Missouri, United States

## Abstract

Alcohol consumption reduces but pain rises over the life course. Thus, we hypothesized that developmental variability in the bidirectional association between alcohol consumption and pain would vary as a function of age. This hypothesis was tested across three age groups – younger (<29), middle (29-65), and older (>65) using NESARC wave 1 and 2 data (N=34,653). The effect of pain interference at baseline on alcohol consumption at follow-up was non-significant across the age groups, indicating that self-medication theory was unsupported. The effect of alcohol consumption at baseline on pain interference at follow-up was significant among the middle (Estimate -.007, p=.002) and older (Estimate -.019, p<.001) groups, but non-significant among the younger group. This latter effect differed significantly between the younger and older groups (p =.005) and the middle and older groups (p=.041). Results show that alcohol consumption reduces pain interference, especially later in life.

